# Significant improvements in InGaN/GaN nano-photoelectrodes for hydrogen generation by structure and polarization optimization

**DOI:** 10.1038/srep20218

**Published:** 2016-02-08

**Authors:** Tao Tao, Ting Zhi, Bin Liu, Mingxue Li, Zhe Zhuang, Jiangping Dai, Yi Li, Fulong Jiang, Wenjun Luo, Zili Xie, Dunjun Chen, Peng Chen, Zhaosheng Li, Zhigang Zou, Rong Zhang, Youdou Zheng

**Affiliations:** 1 Jiangsu Provincial Key Laboratory of Advanced Photonic and Electronic Materials, School of Electronic Science and Engineering, Nanjing University, Nanjing 210093, P. R. China; 2 Ecomaterials and Renewable Energy Research Center (ERERC), School of Physics, Nanjing University, Nanjing 210093, P. R. China; 3 National Laboratory of Solid Microstructures, Collaborative Innovation Center of Advanced Microstructures, Nanjing University, Nanjing 210093, P. R. China; 4 Department of Physics, College of Science, China University of Mining and Technology, Xuzhou, Jiangsu Province 221116, P. R. China

## Abstract

The photoelectrodes based on III-nitride semiconductors with high energy conversion efficiency especially for those self-driven ones are greatly desirable for hydrogen generation. In this study, highly ordered InGaN/GaN multiple-quantum-well nanorod-based photoelectrodes have been fabricated by a soft UV-curing nano-imprint lithography and a top-down etching technique, which improve the incident photon conversion efficiency (IPCE) from 16% (planar structure) to 42% (@ wavelength = 400 nm). More significantly, the turn-on voltage is reduced low to −0.6 V, which indicates the possibility of achieving self-driven. Furthermore, SiO_2_/Si_3_N_4_ dielectric distributed Bragg reflectors are employed to further improve the IPCE up to 60%. And the photocurrent (@ 1.1 V) is enhanced from 0.37 mA/cm^2^ (original planar structure) to 1.5 mA/cm^2^. These improvements may accelerate the possible applications for hydrogen generation with high energy-efficiency.

Photo-electrochemical water splitting for hydrogen generation has recently attracted increasing attentions owing to its store solar energy into chemical energy^1^,^2^,^3^,^4^,^5^,^6^, revealing the possibility to replace current fossil fuel^1^,^2^,^4^. Materials served as photoelectrodes (PEs) for efficient water splitting should fulfill the following requirements: (i) a good match between the absorption wavelength and the solar spectrum to satisfy the energetic for electrolysis; (ii) a high quantum yield; (iii) a correct energy band edge straddling water’s redox potential^2^. So far, various kinds of materials have been extensively investigated as photochemical catalysts, such as Fe_2_O_3_, BiVO_4_, Ta_3_N_5_, etc^2^,^7^,^8^. However, some of them suffer from a low carrier mobility^1^,^9^,^10^, which leads to the suppression of incident photon conversion efficiency (IPCE)^11^. BiVO_4_-based PEs have to work with the assistant of promoter under a high applied external bias^12^,^13^. The surface of Ta_3_N_5_ photoelectrodes is easy to be oxidized, which is not stable enough for future applications^3^. Very recently, III-nitride semiconductors are considered as one of the most suitable candidate materials for solar hydrogen production^14^,^15^,^16^,^17^, especially the indium gallium nitride (InGaN) alloys, which have an adjustable direct band gap perfectly matching solar spectrum, a high carrier mobility and excellent chemical stability^18^,^19^,^20^.

In spite of these potential properties, III-nitride based photoelectrodes still have several problems to be solved in advance^19^,^21^,^22^,^23^. For instance, n-type GaN with the band gap of 3.4 eV and moderate crystal quality, can merely work under the ultraviolet (UV) illumination^21^,^24^. The high density dislocations and strong polarization effect in InGaN layer will increase leakage and suppress the performance of the devices. To resolve above impacts, recent efforts on GaN based photoelectrodes particularly using electrochemical treatment of material surface or active layer doping has been reported^15^,^19^,^25^; however, the efficiency of the hydrogen generation is still challenging due to unprohibited polarization effect. The InGaN-based PE still has huge potential for further improvement.

Basically, the conversion efficiency are strongly affected by the absorption of semiconductor materials, depending on their alloy compositions and thicknesses^15^,^23^. In our previous report, when the thickness of In_0.2_Ga_0.8_N was increased from 60 to 250 nm, the IPCE at the wavelength of 400 nm had been greatly enhanced to 42%^15^. However, for InGaN layer grown on GaN substrate, the further increase of thickness will generate more defects due to the lattice mismatch between InGaN and GaN^26^,^27^,^28^,^29^,^30^. Alternatively, InGaN/GaN multiple quantum wells (MQWs) have been employed^31^ since the pseudomorphic growth thickness can be achieved more than 200 nm with MQWs periods over 15. However, it exists strong piezoelectric polarization along the [0001] growth direction (c-axis), which can cause high level polarization electric field (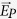)^21^,^26^,^32^,^33^. In terms of InGaN/GaN MQWs, the vector of 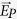 is anti-parallel to the built-in electric field 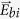, consequently weakening the ability of separating photon-generated carriers and increasing the turn-on voltage as well. As to enhance the photochemical reaction and relieve the polarization-induced electric field, firstly, high crystalline quality InGaN/GaN MQWs were grown on patterned c-plane sapphire substrates (PSS) in this work. Then, the periodic InGaN/GaN nanorods were fabricated by nanoimprint lithography (NIL) and top-down etching fabrication process^34^. As a consequence, the lowest turn-on voltage of the fabricated nano-photoelectrode (nano-PE) device has been obtained down to ~ −0.6 V (with respect to reversible hydrogen potential, RHE, see Methods), which indicates the nano-PE is able to generate hydrogen by itself. Furthermore, SiO_2_/Si_3_N_4_ dielectric distributed Bragg reflectors (DBRs) deposited at the backside greatly can enhance the re-absorption of MQWs, which is benefit to the improvement of conversion efficiency. And the photocurrent (@ 1.1 V vs. RHE) of nano-PE with DBR structures is significantly enhanced from 0.37 mA/cm^2^ (original planar structure) to 1.5 mA/cm^2^. The best value of IPCE (@ 400 nm wavelength) can be achieved as high as 60%.

## Results

### Sample design and fabrication

As designed, 15-fold of InGaN (3 nm)/GaN (11 nm) MQWs were grown on PSS. In content of In_x_Ga_1-x_N layer in MQWs is 20%. The total thickness of MQWs is beyond 200 nm. It should be emphasized that the utilization of PSS not only provides high crystalline quality for epitaxial structures^35^,^36^, but also improves PE’s re-absorption (discuss later). Details including the fabrication of the plane/nano-PE, as well as measurement and simulation methods for this report can be referred to the Methods.

After growth, highly periodic InGaN/GaN nanorod structures were fabricated using the soft UV-curing NIL and top-down etching^34^ (see Methods). A number of advantages of nanorod structures need to be emphasized for photoelectrochemical reaction: 1) it is able to increase the absorption of active area by reducing surface reflection^18^ (see Supplementary Fig. S2); 2) the carrier lifetime can be prolonged due to strain relaxation, which indicates the carrier diffusion length *L*_*diff*_ will be enhanced, further increasing the number of carriers involved in photoelectrolysis^32^; 3) nanorod structures can effectively shorten the distance of carrier transportation^14^,^18^, and enlarge the surface-to-volume ratio, as to enhance the photoelectrochemical reaction^4^. In this work, the optimized uniform diameter of nanorods is approximately 200 nm below the intrinsic *L*_*diff*_ (200–300 nm) for InGaN/GaN nanorods^32^.

**Figure 1:**
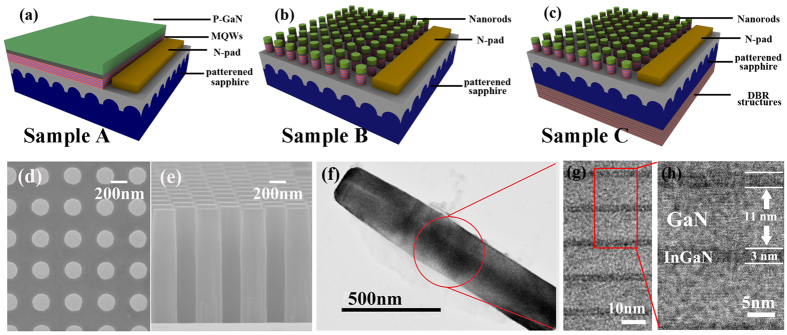
Device structure. Schematics of (a) planar-PE (sample A), (b) nano-PE (sample B), (c) nano-PE with DBR structures (sample C), respectively. (**d**) Top view SEM image of sample surface, and (**e**) side view SEM image of nanorod structures. (**f**) The cross-section TEM image of single nanorod, (**g**) amplified TEM image of MQWs, and (**h**) high resolution TEM image of MQWs.

Figure 1(a–c) show the schematics of sample A, B and C, respectively. Sample A is typical InGaN/GaN MQWs PE with planar structure, which is similar to ordinary LEDs. Sample B is nano-PE with highly ordered nanorods structure. Sample C is nano-PE with additional DBR structures. As shown in Fig. 1(d,e), the SEM images confirm the nanorods are uniformly 250 nm in diameter, 1400 nm in length and with center-to-center separation of 400 nm. The cross-section transmission electron microscope (TEM) image in Fig. 1(f) indicates uniform cylindrical shape with nearly vertical smooth sidewall by the way of KOH wet-etching process. The smooth sidewall is helpful for reducing leakage current and surface corrosion^15^,^37^. As shown in high resolution TEM images of InGaN/GaN MQWs in Fig. 1(g,h), the thickness of each layer is consistent with our design. The active MQWs layers for PE have uniform thicknesses and clear interface between wells and barriers, which indicates the good quality of MQWs.

### DBR structures

**Figure 2:**
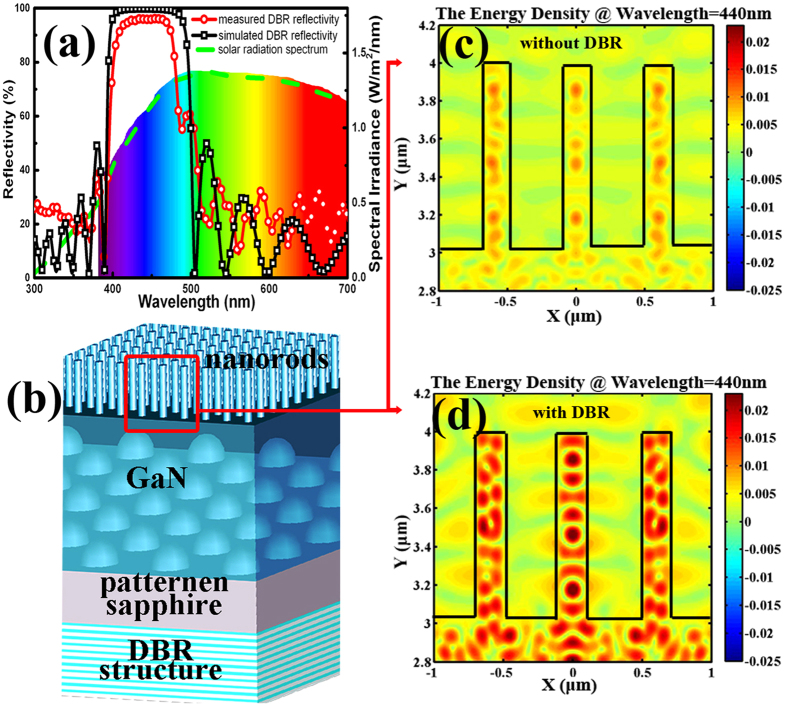
Characteristics of DBR structures. (**a**) Measured and simulated reflectance spectra of 12.5-pairs SiO_2_/Si_3_N_4_ DBR structures, where the background indicates the corresponding color. (**b**) Schematic of nano-photoelectrode with DBR structures for FDTD simulation. (**c**,**d**) show the simulated energy density distribution in nano-photoelectrode without/with DBR structures in the X-Z plane, respectively.

After the fabrication of nanorods, the InGaN/GaN MQWs volume will be reduced. The light will transmit through the gap of nanorods without being absorbed, which might suppress the absorption efficiency. Therefore we design an additional 12.5 pairs SiO_2_/Si_3_N_4_ DBR structures at the backside of sapphire act as a mirror to produce re-absorption process. These dielectric DBR structures can offer high reflectivity as well as strong resistance to acid, alkali and salt solution, which is superior to metal mirrors^38^. The design of DBR structures is based on the optical transfer-matrix approach to calculate the reflectivity band in order to match the absorption of PE (see Methods)^39^. As design, the high reflectivity region should cover the wavelength ranging from 400 nm to 500 nm, corresponding to the color from violet to green. The measured reflectance spectra of DBR structures (red circle curve in Fig. 2(a)) show high reflectivity up to ~95% (calibrated by aluminum mirror), which is consistent with design (black square curve in Fig. 2(a)).

In order to verify the effect of the designed DBR structures, the finite difference time domain (FDTD) simulations are adopted to illustrate the electromagnetic energy density distribution in sample B and C. Schematic of PE device is shown in Fig. 2(b), where the red box indicates the investigation region. As shown in Fig. 2(c,d), the density of electromagnetic energy distribution for sample C at the wavelength of 440 nm is located in the nanorods, nearly 2 times higher than that for sample B, which indicates the re-absorption process is definitely enhanced by utilizing DBR structures.

### Photoelectrochemical measurements

**Figure 3:**
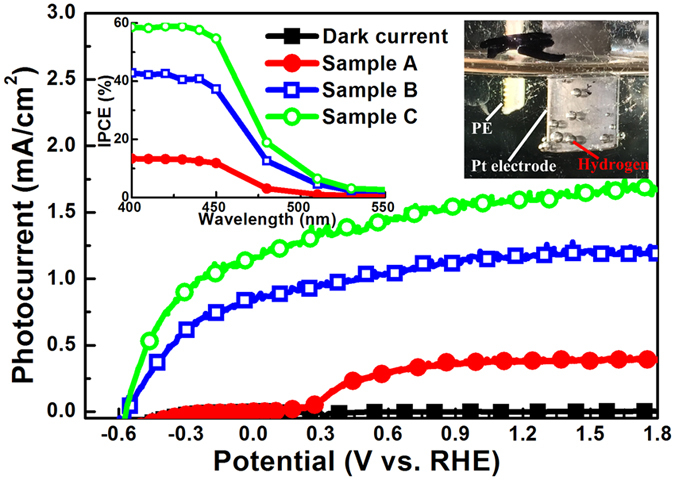
Device photoelectrolysis property. Photocurrent density potential at all samples in 1 M HBr solution under λ > 390 nm illumination. Inset: IPCE of all PE samples in 1 M HBr solution; the potential is 1.1 V vs. RHE. And photo image of hydrogen generation process. Movie of hydrogen generation can be found in the supporting information, movie 1.

The photocurrent and IPCE of sample A, B and C were measured in 1 M HBr aqueous solution^23^, and the results are shown in Fig. 3. The photocurrent of sample B at the potential of 1.1 V (vs. RHE) is significantly enhanced from 0.37 mA/cm^2^ (sample A) to 1.05 mA/cm^2^, which is mainly attributed to the nanorods structures. Moreover, the photocurrent of sample C is further improved to 1.5 mA/cm^2^, which is attributed to the increased re-absorption by additional DBR structures. By the way, the dark current remain quite low even at 1.8 V. It is important to mention that the turn-on voltages (vs. RHE) of sample B and C are unequivocally reduced to ~ −0.6 V, compared to that of sample A at 0.3 V. The reduction in turn-on voltage is mainly attributed to polarization optimization through nanorods fabrication, which will be discussed later. Meanwhile, the photocurrent of sample C can maintain at 1.5 mA/cm^2^ for over 10,000 seconds, which indicates the excellent stability of the fabricated nano-PE. The chronoamperometry (*I*-*t*) as well as the morphology of nano-PE before/after hydrogen generation measurement further prove their good stability (see Supplemental Fig. S5). The hydrogen generated at Pt electrode can be clearly seen in the inset of Fig. 3. Hence, it can be concluded that the fabrication of nano-PE can not only significantly improve the energy conversion efficiency but also reduce the turn-on voltage, which is critical element for applications in hydrogen production.

The IPCE of all samples were also measured in 1 M HBr electrolyte with the electrode potential at 1.1 V vs. RHE (see inset in Fig. 3) by the following equation (1):


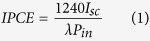


where *I*_*sc*_ is the current density, *λ* is the wavelength of incident light, and *P*_*in*_ is the incident light intensity^15^,^23^,^29^. As shown in the inset, the IPCE of sample A (@wavelength = 400 nm) is obtained to be 14%, while the IPCE of sample B is greatly enhanced to be 42% after fabrication of nanorods. The IPCE of sample C is further enhanced to around 60% with additional DBR structures. Normally, the IPCE of PE can be theoretically estimated by the combination of surface reflection, absorption efficiency, and internal quantum efficiency. According to our previous experimental results^15^, the FDTD simulations (see Supplementary Fig. S1)^40^ and the estimated internal quantum efficiency (IQE) (see Supplementary Fig. S3)^41^,^42^,^43^, the IPCE (@ wavelength = 400 nm) is estimated to be 75~79%. The fabricated nano-PE acquires as much as 60% IPCE with the assistant of DBR structure, which is quite close to the estimated value, indicating outstanding device performance. We believe some other mechanisms, such as Auger recombination^44^, surface recombination^45^ and radiative recombination^32^ should also be responsible for the efficiency reduction^46^,^47^,^48^.

## Discussion

**Figure 4:**
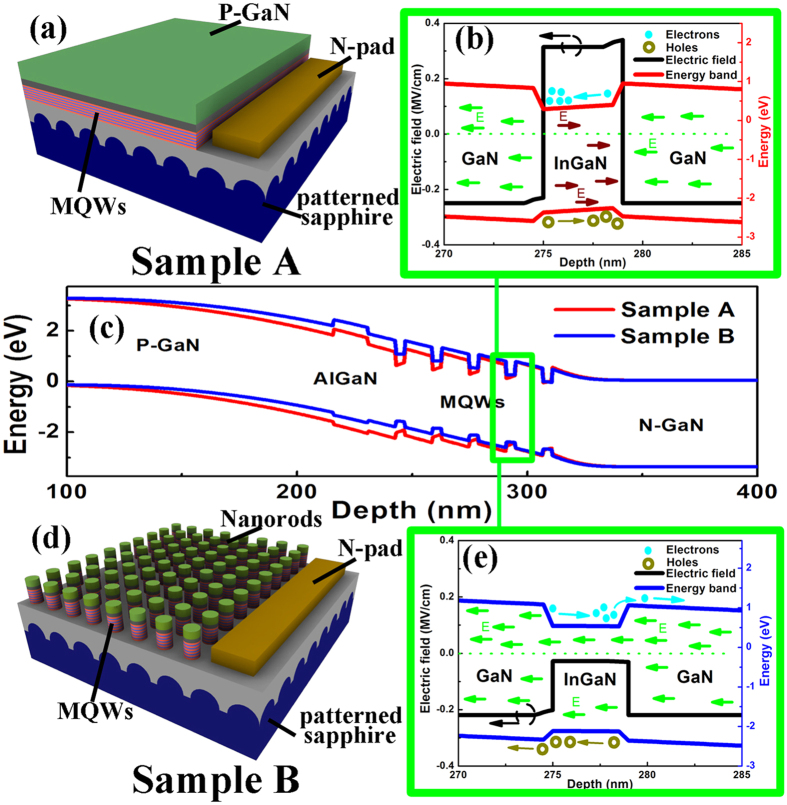
Polarization-induced device. (**a**) Schematics of planar sample A. (**b**) Amplified energy band diagram and electric field of single QW in sample A, where cyan (brown) balls indicate the electrons (holes) and green/wine arrows indicate the electric field in GaN/InGaN. (**c**) The representative energy band diagrams of 5 periods MQWs sample A and B simulated by Silvaco Atlas software. (**d**) Schematics of nanorods sample B. (**d**) Amplified energy band diagram and of electric field single QW in sample B, where cyan (brown) balls indicate the electrons (holes) and green arrows indicate the electric field.

There are several factors could lower the turn-on voltage of PE by forming nanorods structures. First of all, the reduction in polarization effect in InGaN/GaN based PE should take the most responsibility. In addition, the surface area is increased about 3.5 times compared to planar structure. And the nanorod structure can expose other non-polar crystal facets such as m-plane and a-plane. As known, there is an uniquely strong polarization in wurtzite III-nitride semiconductor based heterostructures^32^,^49^. The polarization *P* contains two parts: spontaneous polarization (*P*_*SE*_) and piezoelectric polarization (*P*_*PE*_). *P*_*SE*_ comes from the lack of inversion symmetry in III-nitride (0001) plane^50^, which is hard to avoid. On the other hand, InGaN/GaN heterostructures grown under strain would lead to *P*_*PE*_, which is normally stronger than *P*_*SE*_^51^. The polarization will produce polarization-induced electric field *E*_*P*_, which is parallel to the [000–1] direction against to the built-in electric field *E*_*bi*_ of p-n junction (along the [0001] growth-direction, c-axis). The *E*_*P*_ existed in InGaN/GaN MQWs can be as high as several MV/cm^52^,^53^,^54^,^55^,^56^, close to the value of p-n junction. It could reduce the *E*_*bi*_, consequently suppressing the capability of separating photon-generated carriers. In order to understand the effect of reduced polarization, the energy band diagram and electric field simulations (see Methods)^57^ sample A and B are shown in Fig. 4(c). Figure 4(b) is amplified energy band diagram and electric field of single QW in sample A. There is an energy barrier at the interface between InGaN and right (lower) GaN layer. The electric field *E* in InGaN well layer is estimated to be 0.3 MV/cm, whose direction is opposite to the c-axis (shown as brown arrows). It will make electrons accumulate to the left (upper) GaN barrier layer, which is not benefit for electrons collection and will reduce the photovoltage. The most key issue is to relax the strain accumulated within the InGaN/GaN MQWs in order to get the *P*_*PE*_ reduced. To achieve that, our approach is to fabricate the planar InGaN/GaN MQWs (as shown by Fig. 4(a)) into nanorod structures (as shown by Fig. 4(d)). The measured reciprocal space mappings (RSMs) of InGaN/GaN nano-PE (see Supplementary Fig. S4) indicate as much as 70% strain relaxation^55^,^56^. The strain relaxation in nano-PE can effectively reduce the piezoelectric polarization therefore lowering the polarization-induced electric field *E*_*P*_. As illustrated in Fig. 4(e), the electric field in InGaN well layer become along with c-axis, which can make the electrons (holes) move towards to n-GaN (p-GaN) layer, therefore increase the photovoltage. The measured photocurrent of nano-PE is enhanced by 3 times, and the turn-on voltage is significantly reduced from 0.3 V to −0.6 V (vs. RHE). It is also observed that sample C can obtain photocurrent of 1.1 mA/cm^2^ at zero bias, which is essential for self-driven applications^58^. In order to further confirm the effect of reduced polarization, both planar and nanorod structures based p-GaN/i-In_0.2_Ga0_.8_N/n-GaN PE are comparatively investigated, similar enhancement in photocurrent and reduction in turn-on voltage have also been found (see supplemental Fig. S6).

## Conclusions

In summary, the energy conversion efficiency of InGaN/GaN photoelectrodes has been greatly enhanced through the fabrications of highly ordered nanorods and DBR structures. Consequently, the photocurrent of nano-PE with ordered nanorods structure and additional DBR structures is enhanced up to 1.5 mA/cm^2^ (@ 1.1 V vs. RHE) and the IPCE is improved to be about 60% under the visible light irradiation. It is of importance that the turn-on voltage of nano-PE is effectively reduced to around −0.6 V through polarization modulation. Under the zero bias, nano-PE can obtain the photocurrent of 1.1 mA/cm^2^, which is vital for self-driven applications. Those enhanced characteristics bring bright future for nitride semiconductor based devices for hydrogen generation.

## Methods

### Epitaxial growth of InGaN/GaN MQWs on patterned sapphire substrates

The InGaN/GaN MQWs used in this study were grown on patterned c-sapphire (0001) substrates by metal-organic chemical vapor-phase deposition (MOCVD), as to achieve high crystalline quality. Trimethyl-gallium (TMGa), Trimethyl-indium (TMIn), and ammonia (NH_3_) were the source precursors for Ga, In and N, respectively. The sample structure is similar to a standard InGaN/GaN LED structure, which consists of 2 μm undoped GaN buffer layer, followed by 2 μm silicon doped n-type GaN, a thin InGaN strain released layer, 15 periods (In_0.2_Ga_0.8_N:3 nm/GaN:11 nm) MQWs. A thin Al_0.25_Ga_0.75_N with 25 nm was used as an electron blocking layer (EBL). Finally, the sample was capped with 500 nm p-type GaN.

### Nanorods fabrication

Highly ordered nanorods were fabricated by the developed soft ultraviolet (UV)-curing nanoimprint lithography (NIL) and top to bottom etching technique^34^. At first, 200 nm thick silicon dioxide (SiO_2_) grown by plasma-enhanced chemical vapor deposition (PECVD) was used as protection layer. Spin coated PolymethylMethacrylate (PMMA) and UV resist were adopted to form the ordered nano-patterns. After that, a 20 nm thick nickel mask was deposited on the surface by physical vapor deposition (PVD). Periodic nickel islands were formed by lift-off process. Ion etching (RIE) process and standard inductively coupled plasma (ICP) etching were applied to form nanorods with a height of approximately 1.4 μm. And 1 mol/L thermal KOH solution was employed to treat the nanorods for curing the etching damage.

### SEM and TEM imaging

The scanning electron microscope (SEM) images of nanorod structures were performed with a field emission SEM (JEOL JSM-7000F). The electron beam with acceleration voltage of 10 kV and current of 8 nA were applied. Nanorods were mechanically removed from the substrate and ultrasonicated in alcohol solution. A few drops of the solution were then put on a carbon-coated TEM grid. After evaporation of the liquid, the cross-sectional TEM images of single nanorod were acquired by a FEI Tecnai F20 TEM. All measurements were done at room temperature.

### DBR structure fabrication

The SiO_2_/Si_3_N_4_ dielectric DBR structures were designed by the optical transfer-matrix approach, whose reflection zone was targeted within the wavelength range from 400 to 500 nm as to fitting the absorption region of InGaN/GaN-based photoelectrodes. The DBR structures were grown by PECVD. Before the deposition, mechanical polishing process was applied at the backside of sapphire to form smooth surface. The reflectance spectra of DBR structures were gathered by UV-visible spectrometer calibrated by aluminum mirror.

### Device fabrication and measurement

For both the planar and nanorod photoelectrode device, as to collect photon-generated electrons, the n-type pad was patterned by photolithography, and then etched down to the n-GaN layer by ICP, where multi-layer metals of Ti/Al/Ni/Au with thicknesses of 10 nm/100 nm/10 nm/100 nm were deposited and annealed as to form ohmic contact.

The photoelectrolysis of all photoelectrodes were measured by three-electrode cell using an electrochemical analyzer (CHI-633C, Shanghai Chenhua) under the light with different wavelengths generated by monochrome filters. The light intensity was measured with a photometer (Newport, 840-C). The photocurrent and IPCE of nanorod photoelectrode with/without DBR structures were measured in 1 M HBr aqueous solution. A 500 W xenon lamp was employed as light source (100 mWcm^−2^). As to exclude the ultraviolet light, a 390 nm cutoff filter was employed to make sure the wavelength of light source λ > 390 nm. The samples, a Pt wire, and a saturated calomel electrode (SCE) were used as working, counter and reference electrodes, respectively. The potentials vs. SCE have been changed into that vs. RHE. The potentials of the working electrode were shifted at a RHE (reversible hydrogen potential) scale by the formula V_RHE_ = V_SCE_ + 0.242V + 0.059 × pH, where V_RHE_ was the potential vs. reversible hydrogen potential, V_SCE_ was the potential vs. SCE reference electrode, and pH was the pH value of electrolyte.

### Silvaco Atlas software simulation

Silvaco Atlas software was adopted in order to illustrate the effect of polarization-induced energy band diagram of the samples. The hole concentration for the p-type GaN layer is 5 × 10^17^ cm^−3^, and electron concentration is 2 × 10^18^ cm^−3^ for the n-type layer, respectively. The indium composition of the MQWs was set to 20%, the same as that in the grown sample. In the simulations, the drift-diffusion model using K•P band parameters, Fermi-Dirac statistics model and incomplete ionization model were used. The carrier generation-recombination process consists of terms including concentration-dependent lifetime Shockley-Read-Hall (CONSRH), radiative recombination, Auger recombination and optical generation-recombination. Additionally, the polarization effect of quantum well layer is enabled by setting the polarization parameters, which are specified 0.5 for planar and 0.15 for nanorod samples.

## Additional Information

**How to cite this article**: Tao, T. *et al.* Significant improvements in InGaN/GaN nano-photoelectrodes for hydrogen generation by structure and polarization optimization. *Sci. Rep.* **6**, 20218; doi: 10.1038/srep20218 (2016).

## Supplementary Material

Supplementary Information

Supplementary Video 1
