# Supplementary material for: Significant improvements in InGaN/GaN nano-photoelectrodes for hydrogen generation by structure and polarization optimization
Source: Sci Rep. 2016 Feb 8;6:20218. doi: 10.1038/srep20218 (PMC4745013; doi:10.1038/srep20218)
Supplement: Supplementary Information [file srep20218-s1.doc]

# Supporting materials

**Significant improvements in InGaN/GaN nano-photoelectrodes for hydrogen generation by structure and polarization optimization**

Tao Tao^1,3^,Ting Zhi^1,3^, Bin Liu^1,3ξ^, Mingxue Li^2,3,4^, Zhe Zhuang^1,3^,Jiangping Dai^1,3^, Yi Li^1,3^, Fulong Jiang^1,3^, Wenjun Luo^2,3^, Zili Xie^1,3^, Dunjun Chen^1,3^, Zhaosheng Li^2,3^, Zhigang Zou^2,3^, Rong Zhang^1,3ξ^, and Youdou Zheng^1,3^

^1^*Jiangsu Provincial Key Laboratory of Advanced Photonic and Electronic Materials,* *School of Electronic Science and Engineering, Nanjing National Laboratory of Microstructures, Nanjing University, Nanjing 210093, P. R. China*

^2^ *Ecomaterials and Renewable Energy Research Center (ERERC), Department of Physics, Nanjing University, Nanjing 210093, People’s Republic of China*

^3^*National Laboratory of Solid Microstructures, Nanjing University, Nanjing 210093, People’s Republic of China*

*^4^Department of Physics, College of Science, China University of Mining and Technology, Xuzhou, Jiangsu Province 221116*

^ξ^Corresponding authors: [bliu@nju.edu.cn](mailto:bliu@nju.edu.cn) and [rzhang@nju.edu.cn](mailto:rzhang@nju.edu.cn)

**Absorption of InGaN/GaN MQWs**

The finite difference time domain method (FDTD solutions from Lumerical Solutions, Inc.) was applied to estimate the light absorption of 15-flod InGaN/GaN MQWs structure. The thicknesses of InGaN and GaN layer are 3.5 nm and 12 nm, respectively. The absorption coefficient of InGaN material used in this study is ~5×10^5^ /cm. The wavelength of incidence light is set at 400 nm. One can be seen that the transmissivity gradually decreases from over 80*%* to less than 5*%* with the increasing thickness in Fig. S1. It indicates that the thickness over 200 nm could absorb enough amount of visible light, which is crucial element for photoelectrode device.

The transmissivity of sample A, B and C are shown in Fig. S2 (a). The transmissivity of sample C is obviously lower than those of other samples, which indicates the benefit of DBR structures at backside. The reflectivity of planar and nanorod structures have been measured by grating spectrometer (Zolix Omni-λ 300) and shown as Fig. S2 (b). It can be seen that the nanorods structure can effectively reduce the surface reflection therefore increase the absorption.

**Fig. S1.** Relation between transmissivity and thickness of MQWs simulated by FDTD.


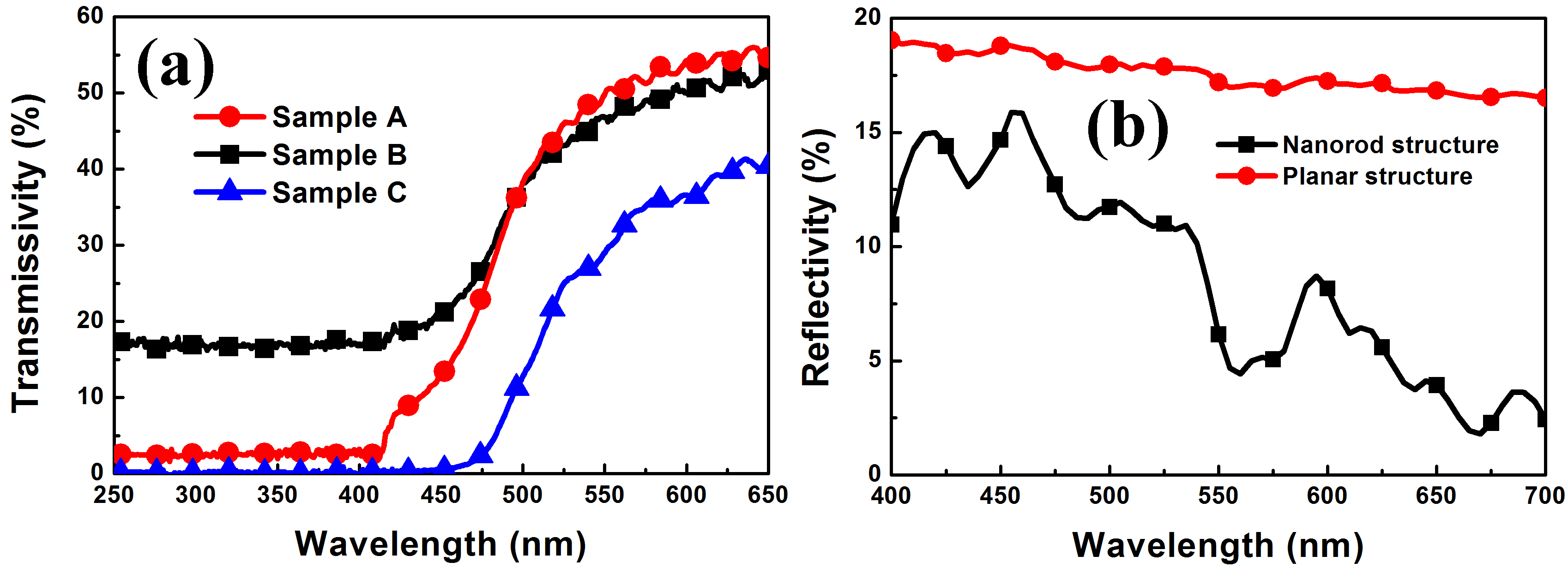


**Fig. S2.** (a) Transmissivity of sample A, B and C. (b) Reflectivity of planar and nanorod structures.

**
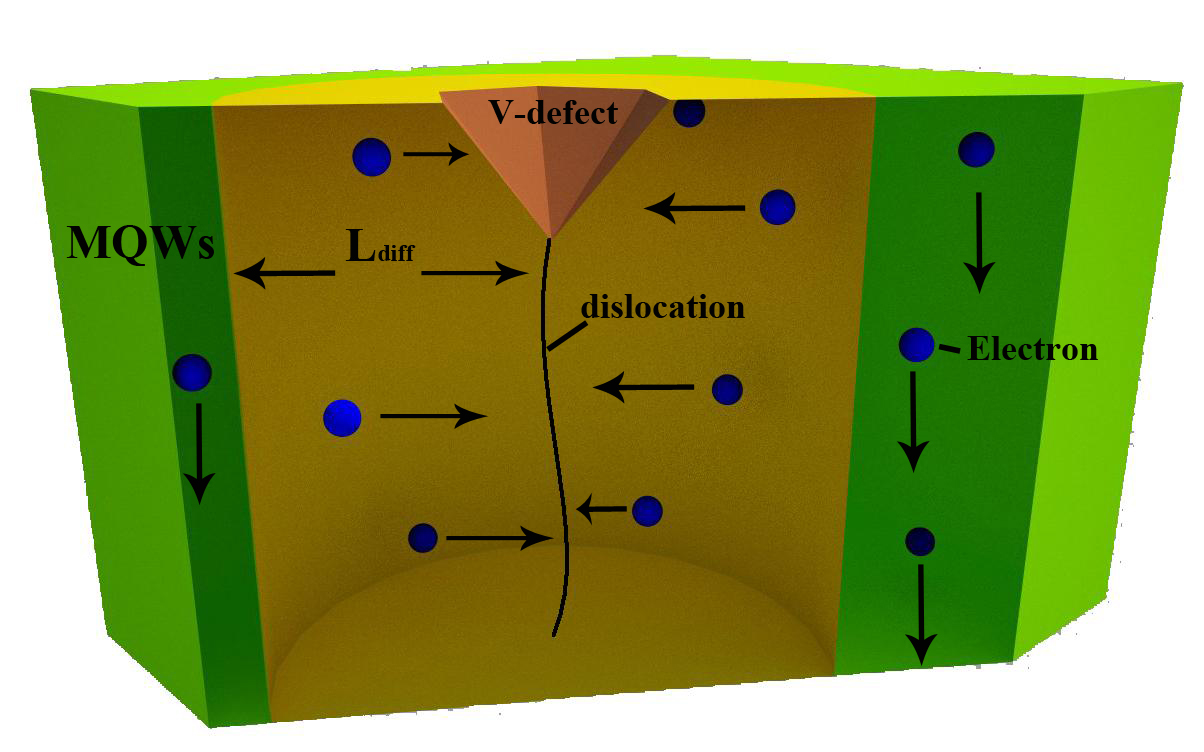
**

**Fig. S3**. Schematic of defect and those electrons affected by dislocation.

**Internal quantum efficiency of PE**

In order to roughly estimate the internal quantum efficiency (IQE) of InGaN/GaN based photoelectrodes, the excitonic diffusion length *L_diff_* and density of V-defect/threading dislocation are taken into account. In order to show the electrons trapping zone, a schematic of defect is illustrated in Fig. S3. The brown inverted hexagonal pyramid indicates the V-defect developed from dislocation, which is normally believed as non-radiative recombination center in InGaN-based hetero-structures. As shown by the yellow cylinder, the photon-generated electrons within this area will be affected by dislocation, and the area is highly related to the excitonic diffusion length *L_diff_*. Thus, the electrons out of this area can be transported to electrodes by electric field. The IQE can be roughly estimated by taking the *L_diff_* (200-300 nm for InGaN/GaN MQWs) and dislocation density (~8×10^7^/cm^2^ from the TEM measurements) in to calculation. The maximum internal quantum efficiency is estimated to be ~88*%*, if some other factors are excluded from consideration.


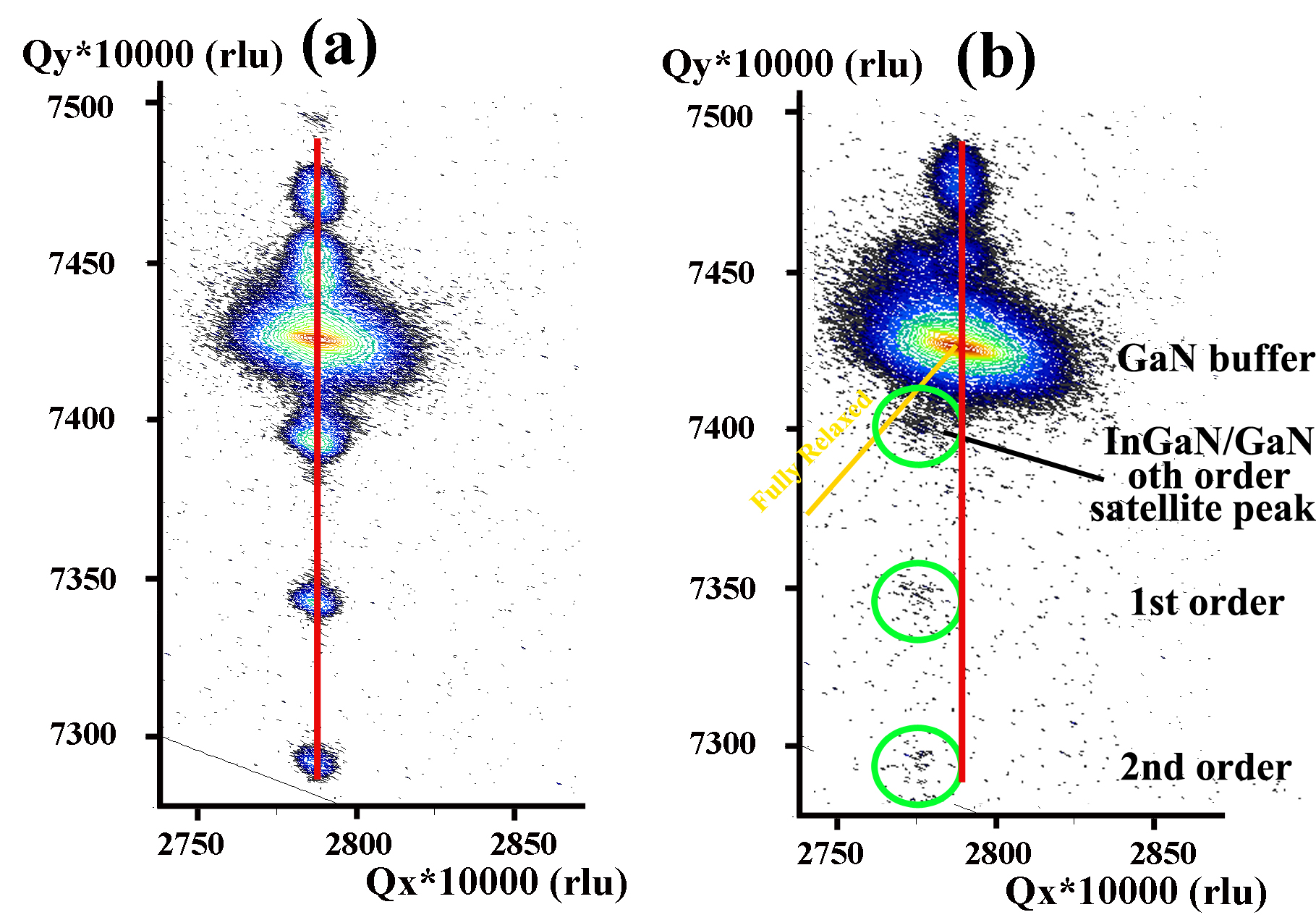


**Fig. S4.** The measured reciprocal space mappings (RSMs) of InGaN/GaN (a) planar-PE and (b) nano-PE.

**The strain relaxation of InGaN/GaN nano-PE**

Normally, there is a strong strain accumulated in InGaN/GaN based heterostructures, especially for the 15-pairs MQWs structures. The planar-PE and nano-PE were measured by PANalytical X’Pert Pro MRD triple-axis X-ray diffraction. As shown in Fig. S4 (a), the satellite peaks of InGaN/GaN in planar-PE share the same Q_x_ as the GaN buffer layer as illustrated by red line. It indicates the InGaN layers in planar-PE are fully strained. On the other hand, the satellite peaks of InGaN/GaN in nano-PE shift towards the yellow line, where the fully relaxed state should be. It confirms that the accumulated strain in InGaN/GaN MQWs can be relaxed by forming nanostructures. Based on the Q_x_ and Q_y_ of the 0th order satellite peak of InGaN/GaN, we can estimate the nano-PE get about 70% strain relaxation[^1-3^](#_ENREF_1).


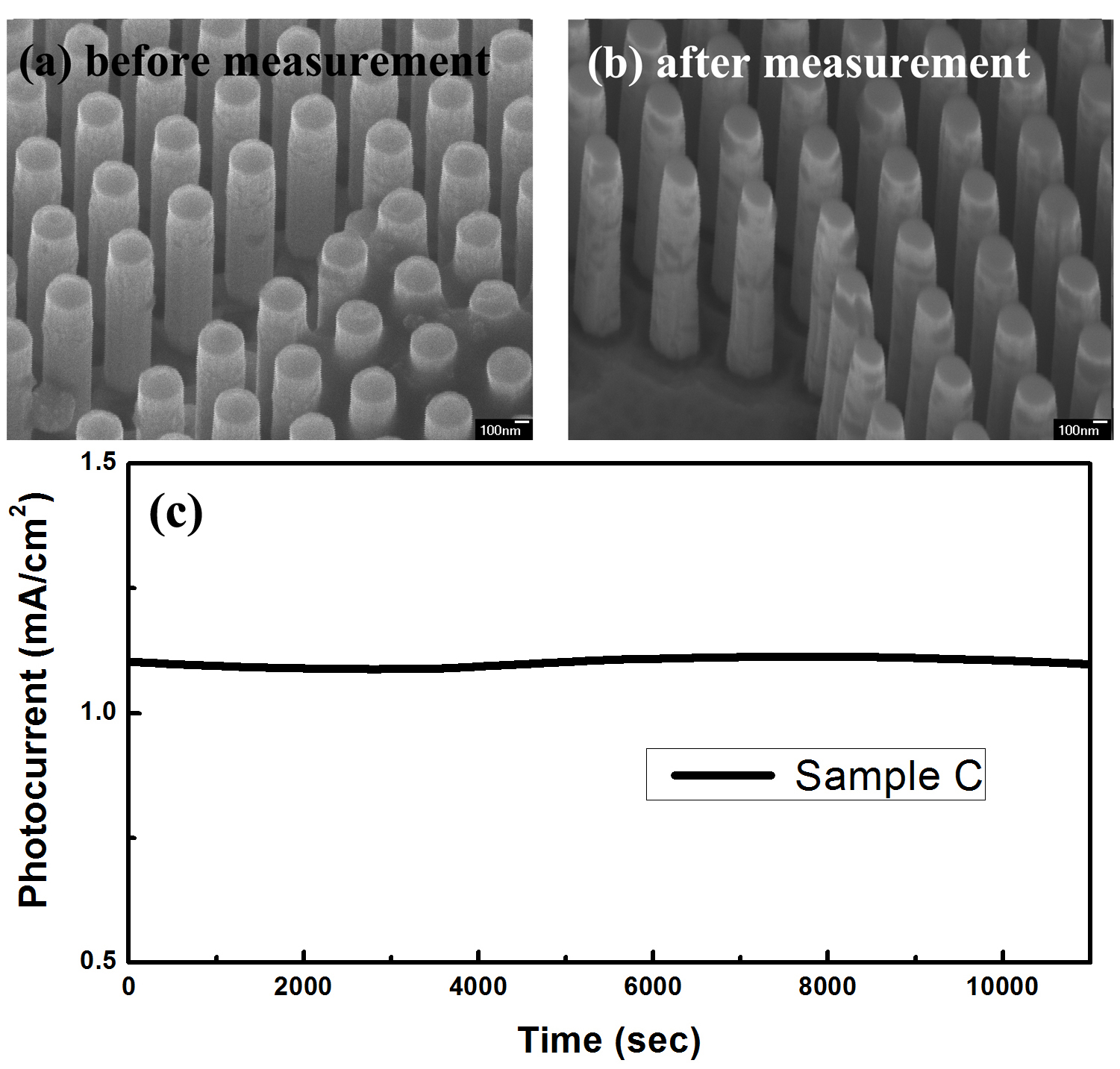


**Fig. S5.** SEM image of sample C before (a) and after (b) long time working. (c) Chronoamperometry (*I-t*) of InGaN/GaN nano-PE.

**Stability of InGaN/GaN nano-PE**

The InGaN/GaN based nano-PE has a good resistance to the acid and alkaline. By comparing the SEM image of nanorods in Fig. S5 (a) with that in Fig. S5 (b), the nanorod structures exhibit barely change after a long time working. Although the nanorods are slightly tapered, the shape is still uniform and sidewalls remain smooth. Our following work will focus on how to reduce the corrosion and further enhance the efficiency with the help of metal co-catalysts.

The chronoamperometry (*I-t*) of InGaN/GaN nano-PE in 1 M HBr aqueous solution under illumination over 390 nm was measured and shown in Fig. S5 (c)[^4^](#_ENREF_4). The photocurrent of sample C remains constant in a long-time (10000s) illumination. It further confirms the strong stability of InGaN/GaN nano-PE.

**InGaN/GaN p-i-n structure fabrication and measurements**

The p-GaN/i-In_0.2_Ga_0.8_N/n-GaN structures involved in this comparative study were grown on c-sapphire (0001) substrates by metal-organic chemical vapor-phase deposition (MOCVD). The sample structure consists of 2 μm undoped GaN buffer layer, followed by 2 μm silicon doped n-type GaN, 200 nm thick InGaN undoped layer, and 200 nm thick p-doped GaN layer. The In content of InGaN layer is 0.2 and confirmed by XRD results.[^5^](#_ENREF_5)

Fig. S6 shows the photocurrent of planar and nanorods samples detected in 1 M HBr aqueous solution. The dark current was measured in dark environment, indicating the leakage; while the photocurrent was measured using a 500 W xenon lamp as light source. The photocurrent (@ 1 V vs. SCE) of nanorods sample is enhanced by about 1.5 times compared to that of planar sample. On the other hand, the polarization field in planar p-i-n structure will greatly reduce the total build-in electric field *E_bi_*. As marked by dot arrow, the turn-on voltage is reduced from 0.5 V (planar sample) down to -0.5 V (nanorods samples), which is attributed to effectively reduced piezoelectric polarization field due to the strain relaxation by forming nanorods.[^6^](#_ENREF_6)


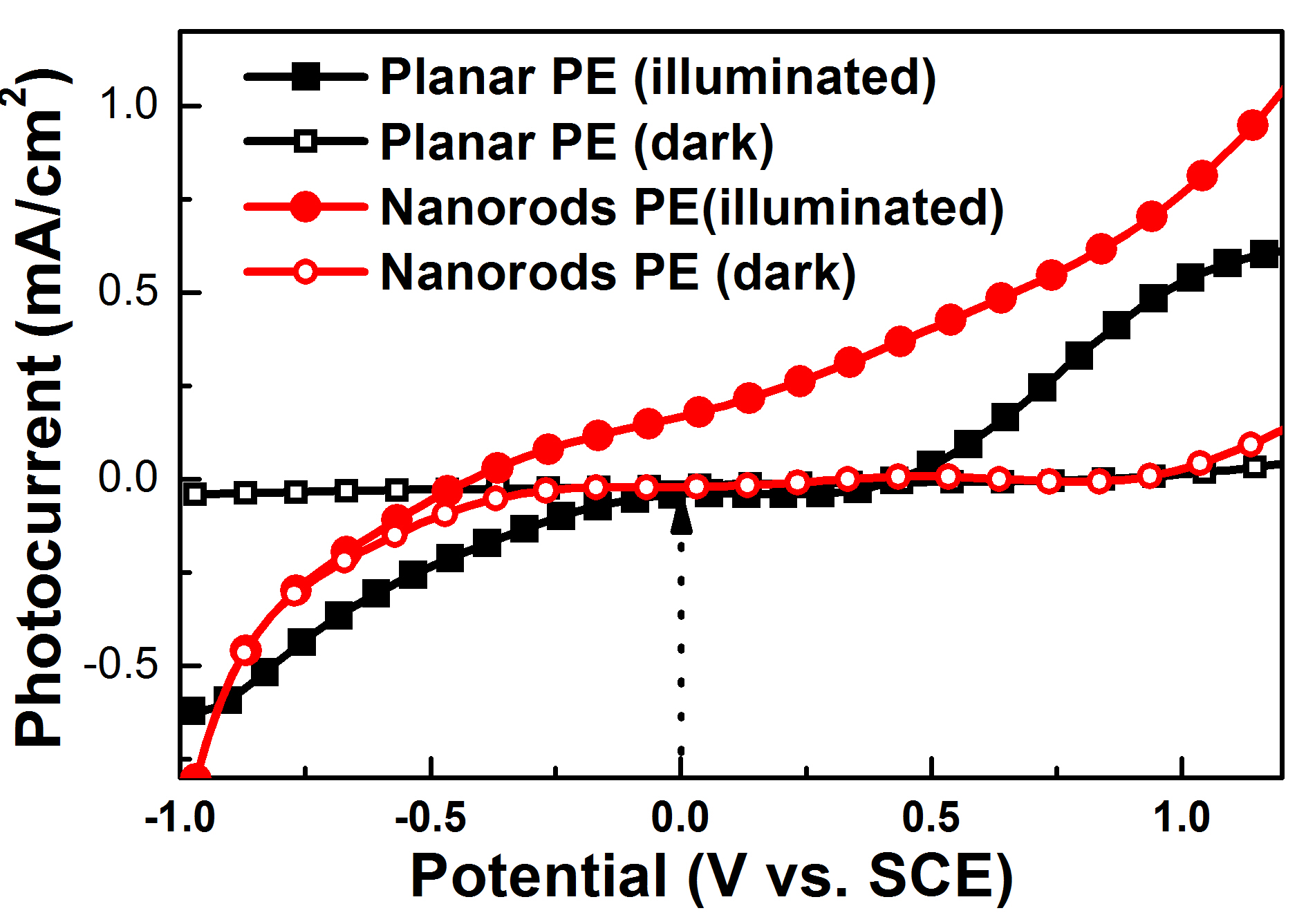


**Fig. S6.** Photocurrent of samples with planar and nanorods structures. Black solid/hollow square curve indicate the photocurrent/dark-current of planar sample, while the red solid/hollow circle curve indicate the photocurrent/dark-current of nanorods sample, respectively.

**References**

1 Moram, M. A. & Vickers, M. E. X-ray diffraction of III-nitrides. *Rep. Prog. Phys.* **72**, 036502 (2009).

2 Li, W. *et al.* High resolution x ray analysis of InGaN/GaN superlattices grown on sapphire substrates with GaN layers. *Appl. Phys. Lett.* **69**, 3390-3392 (1996).

3 Liu, B., Smith, R., Bai, J., Gong, Y. & Wang, T. Great emission enhancement and excitonic recombination dynamics of InGaN/GaN nanorod structures. *Appl. Phys. Lett.* **103**, 101108 (2013).

4 Li, M. X. *et al.* Remarkable enhancement in photocurrent of In_0.20_G_0.80_N photoanode by using an electrochemical surface treatment. *Appl. Phys. Lett.* **99**, 112108-112103 (2011).

5 Liu, B. *et al.* Growth of In-rich and Ga-rich InGaN alloys by MOCVD and fabrication of InGaN-based photoelectrodes. *Phys. Status Solidi C.* **7**, 1817-1820 (2010).

6 Wilsch, B. *et al.* Spatially resolved investigation of strain and composition variations in (In,Ga)N/GaN epilayers. *Appl. Phys. Lett.* **102**, 052109-052104 (2013).
