# Supplementary figures and images for: Significant improvements in InGaN/GaN nano-photoelectrodes for hydrogen generation by structure and polarization optimization
Source: Sci Rep. 2016 Feb 8;6:20218. doi: 10.1038/srep20218 (PMC4745013; doi:10.1038/srep20218)

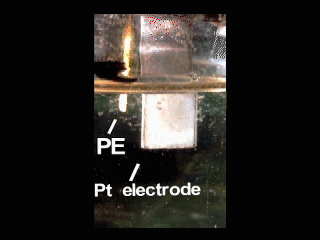

Supplement: Supplementary Video 1 [file srep20218-s2.gif]
